# AVD Best Cited Articles 2020

**DOI:** 10.3400/avd.bca.20-01000

**Published:** 2020-12-25

It is with a great pleasure to announce the top three most-cited AVD articles on Web of Science.

## No. 1

**Special Article**

****An Update on Methods for Revascularization and Expansion of the TASC Lesion Classification to Include Below-the-Knee Arteries: A Supplement to the Inter-Society Consensus for the Management of Peripheral Arterial Disease (TASC II)****

The TASC Steering Committee

Michael R. Jaff, Christopher J. White, William R. Hiatt, Gerry R. Fowkes, John Dormandy, Mahmood Razavi, Jim Reekers, and Lars Norgren

****Citation: 61****

Published: 2015 (Vol. 8 No. 4: 343-357)

## No. 2

**Review Article**

****Visceral Artery Aneurysms and Pseudoaneurysms? Should They All be Managed by Endovascular Techniques?****

Alfredo C. Cordova and Bauer E. Sumpio

****Citation: 51****

Published: 2013 (Vol. 6 No. 4: 687-693)

## No. 3

**Review Article**

****Compression Therapy: Clinical and Experimental Evidence****

Hugo Partsch

****Citation: 50****

Published: 2012 (Vol. 5 No. 4: 416-422)

## Notes

The ranks are based on the citation counts on Web of Science, as of November 10, 2020.

